# Arf1 and Membrane Curvature Cooperate to Recruit Arfaptin2 to Liposomes

**DOI:** 10.1371/journal.pone.0062963

**Published:** 2013-04-29

**Authors:** Ernesto E. Ambroggio, James Sillibourne, Bruno Antonny, Jean-Baptiste Manneville, Bruno Goud

**Affiliations:** 1 Laboratorio de Biofísica de Biomembranas. Centro de Investigaciones en Química Biológica de Córdoba (CIQUIBIC, UNC-CONICET), Departamento de Química Biológica, Facultad de Ciencias Químicas, Universidad Nacional de Córdoba, Córdoba, Argentina; 2 Laboratoire Mécanismes moléculaires du transport intracellulaire, Institut Curie, CNRS UMR 144, Paris, France; 3 Institut de Pharmacologie Moléculaire et Cellulaire, Université de Nice Sophia Antipolis et CNRS, Valbonne, France; Purdue University, United States of America

## Abstract

Arfaptin2 contains a Bin/Amphiphysin/Rvs (BAR) domain and directly interacts with proteins of the Arf/Arl family in their active GTP-bound state. It has been proposed that BAR domains are able to sense membrane curvature and to induce membrane tubulation. We report here that active Arf1 is required for the recruitment of Arfaptin2 to artificial liposomes mimicking the Golgi apparatus lipid composition. The Arf1-dependent recruitment of Arfaptin2 increases with membrane curvature, while the recruitment of Arf1 itself is not sensitive to curvature. At high protein concentrations, the binding of Arfaptin2 induces membrane tubulation. Finally, membrane-bound Arfaptin2 is released from the liposome when ArfGAP1 catalyzes the hydrolysis of GTP to GDP in Arf1. These results show that both Arf1 activation and high membrane curvature are required for efficient recruitment of Arfaptin2 to membranes.

## Introduction

Arfaptin2 (also known as POR1) is a BAR (Bin/Amphiphysin/Rvs) domain protein that was first identified as a Rac1-interacting protein and as a class I Arf (Arf1, 3 and 5) effector, suggesting that it plays an important role in the cross-talk between Arf and Rac-mediated signalling pathways [1]–[4]. Later on, Arfaptin2 was shown to interact with Arl1 (Arf-like protein 1) [5], [6]. Arfaptin2 is also a substrate of the Akt kinase and is involved in neuroprotection in Huntington disease, but whether this function is related to the Arf/Rac pathways remains unclear [7], [8].

Arfaptin2, like other BAR domain-containing proteins, is able to sense membrane curvature and to tubulate liposomes *in vitro* [9]–[12]. *In vivo*, Arfaptin2 is recruited on Golgi membranes by Arl1 and its overexpression induces the formation of tubular structures emanating from the Golgi complex [13], [14]. How BAR domain-containing proteins sense membrane curvature and triggers membrane deformation remains a matter of debate. It has been proposed that the curvature sensing properties of BAR domains are mediated by a higher density of binding sites on curved membranes and not by an increase in the affinity of the protein for the curved membranes [15], [16]. Membrane deformation could be induced by protein-protein crowding at the surface of a membrane, as recently documented for epsin1 and AP180, two proteins involved in the formation of clathrin-coated vesicles [17].

In this study, we have reconstituted Arf1-mediated binding of Arfaptin2 on Large Unilamellar Vesicles (LUVs) and Giant Unilamellar Vesicles (GUVs). We show that Arfaptin2 is recruited by active Arf1 (GTP-bound) and preferentially associates to highly curved membranes. We also provide evidence that Arfaptin2 can trigger membrane tubulation only at high concentration.

## Materials and Methods

### Reagents

Lipids and biotinylated 1,2-Dioleoyl-*sn*-Glycero-3-Phosphoethanolamine-N-(Cap Biotinyl) (Biot-CAP-DOPE) were purchased from Avanti Polar Lipids. The fluorescent *N*-((4-(4, 4-difluoro-5-(2-thienyl)-4-bora-3a, 4adiaza-*s*-indacene-3-yl) phenoxy) acetyl) sphingosine (BODIPY-TR-ceramide, BodTRCer), *N*-(6-tetramethylrhodaminethiocarbamoyl)-1,2-dihexadecanoyl-*sn*-glycero-3-phosphoethanolamine, triethylammonium salt (TRITC-DHPE) and Alexa^488^-C5-Maleimide were from Invitrogen. ATP, GTP, GDP and GTPγS were purchased from Roche Molecular Biochemicals. Streptavidin was obtained from Pierce. The Ni-NTA superflow column was from QIAGEN. All other chemicals were purchased from Sigma Aldrich.

### Protein Purification

Detailed protocols for Arf1 and ArfGAP1 purification have been published previously in Manneville et al. [18] and Ambroggio et al. [19]. The hexa-histidine tagged version of the biotinylated kinesin 1 was purified as described by Thoresen and Gelles [20].

For fluorescence experiments in cuvette, Arf1 tagged with hexa-histidine (Arf1-H6) was purified following the protocols described by Liu et al. [21] and Ha et al. [22]. Briefly, the sequence of Arf1 was cloned into a pET21b vector and expressed in BL21 bacteria. Non-myristoylated and myristoylated Arf1 were co-purified using a Ni-NTA affinity column. The myristoylated version was purified from the non-myristoylated by FPLC using a HP-phenylsepharose column.

Full-length human Arfaptin2 was expressed as an N-terminal His-tag fusion protein in *Escherichia coli* BL21 cells (Stratagene, Agilent Technologies). The cDNA of Arfaptin2 was acquired from Imagenes (Clone IRALp962G021Q), amplified by PCR incorporating the restriction sites *Nde I* (5′; primer AAAAAACATATGACGGACGGGATCCTAGG) and *Xho I* (3′; primer AAAAAACTCGAGTCACTGCTCCTCTAGCCAGG) and cloned into a pET16b vector (Novagen). The protein was purified using a Ni-NTA affinity column. The eluted protein (elution buffer: 50 mM NaH_2_PO_4_, 300 mM NaCl, 300 mM Imidazole) was then incubated with 1 mM DTT for 2 hrs. DTT was eliminated using gel filtration and Alexa^488^ labelling was conducted. The excess dye was eliminated by gel filtration using 20 mM Tris, 150 mM NaCl. The dye/protein ratio was around 2. Finally, the protein (16 µM) was snap-frozen and stored at −80°C.

### Liposome Preparation

Large unilamellar vesicles (LUVs) of a lipid composition similar to Golgi membranes (Golgi-mix: Egg-PC 50%, Liver-PE 19%, Brain-PS 5%, Liver-PI 10% and Cholesterol 16%) were prepared by sequential extrusion through polycarbonate filters of 200 nm, 100 nm, 50 nm and 30 nm pore size in HKM buffer (HEPES 50 mM, KAcetate 120 mM, MgCl_2_ 1 mM) [23], [24]. The final lipid concentration obtained was 4mM. For the FRET experiments 1% mole of fluorescent TRITC-DHPE was added to the lipid mix.

Golgi-mix giant unilamellar vesicles (GUVs) containing 1% mole of fluorescent BODIPY-TR-Ceramide (BodTRCer) and 1% of Biot-CAP-PE were generated by the electroformation technique on ITO (indium-tin-oxide) coverslides [25] in a sucrose solution matching the osmolarity of the used buffers.

### Flotation Experiments

Proteins (0.75 µM) and liposomes (0.75 mM) were incubated in HKM buffer at room temperature for 20 min in a total volume of 150 µl. The suspension was adjusted to 30% sucrose by adding and mixing 100 ml of a 75% w/v sucrose solution in HKM buffer. The resulting high-sucrose suspension was overlaid with 200 µl HKM containing 25% w/v sucrose and 50 µl HKM containing no sucrose. The sample was centrifuged at 55000 r.p.m. (240,000 g) in a Beckman swinging rotor (TLS 55) for 1 h. The bottom (250 µl), middle (150 µl) and top (50 µl) fractions were manually collected from the bottom using a Hamilton syringe and analysed by SDS–PAGE before and after staining with Sypro orange (Molecular Probes). Quantification was performed using the ImageJ software. For each condition (GDP or GTP) the sum of the band signal recovered in the bottom and top fractions was taken as 100% (total protein mass).

### Fluorescence Measurements of Arfaptin2 Recruitment to TRITC-LUVs after Arf1 Activation

Measurements were performed in a cylindrical quartz cuvette containing 0.5 µM Arf1-H6 (initially in the GDP-bound form), 0.5 µM Arfaptin2-Alexa^488^ and 0.5 mM sequentially extruded Golgi-mix liposomes (containing 1% fluorescent TRITC-DHPE) in HKM buffer. The suspension was stirred with a small magnetic bar. Reagents were sequentially added from stock solutions using automatic pipettes. The Alexa^488^ fluorescence emission was excited at 492 nm and measured at 518 nm. Fluorescence measurements were normalized by the value before GTP addition (t = 500 s).

### Arfaptin2 Recruitment to Golgi-mix Lipid Tubes and GUVs

Lipid tubes were pulled from GUVs using the motor domain of kinesin1 coupled with the bacterial biotin carrier protein as described previously [19]. Briefly, after tube pulling, 1 to 3 µM Arf1 (bound to GDP or GTPγS) and Arfaptin2-Alexa^488^ were incubated with the liposome/tube networks. Confocal fluorescence micrographs were obtained using a Zeiss Meta 510 confocal microscope. Analyses were performed using the ImageJ software.

## Results and Discussion

### Arfaptin2 is Recruited to LUVs by Arf1:GTP in a Curvature Dependent Manner

**Figure 1:**
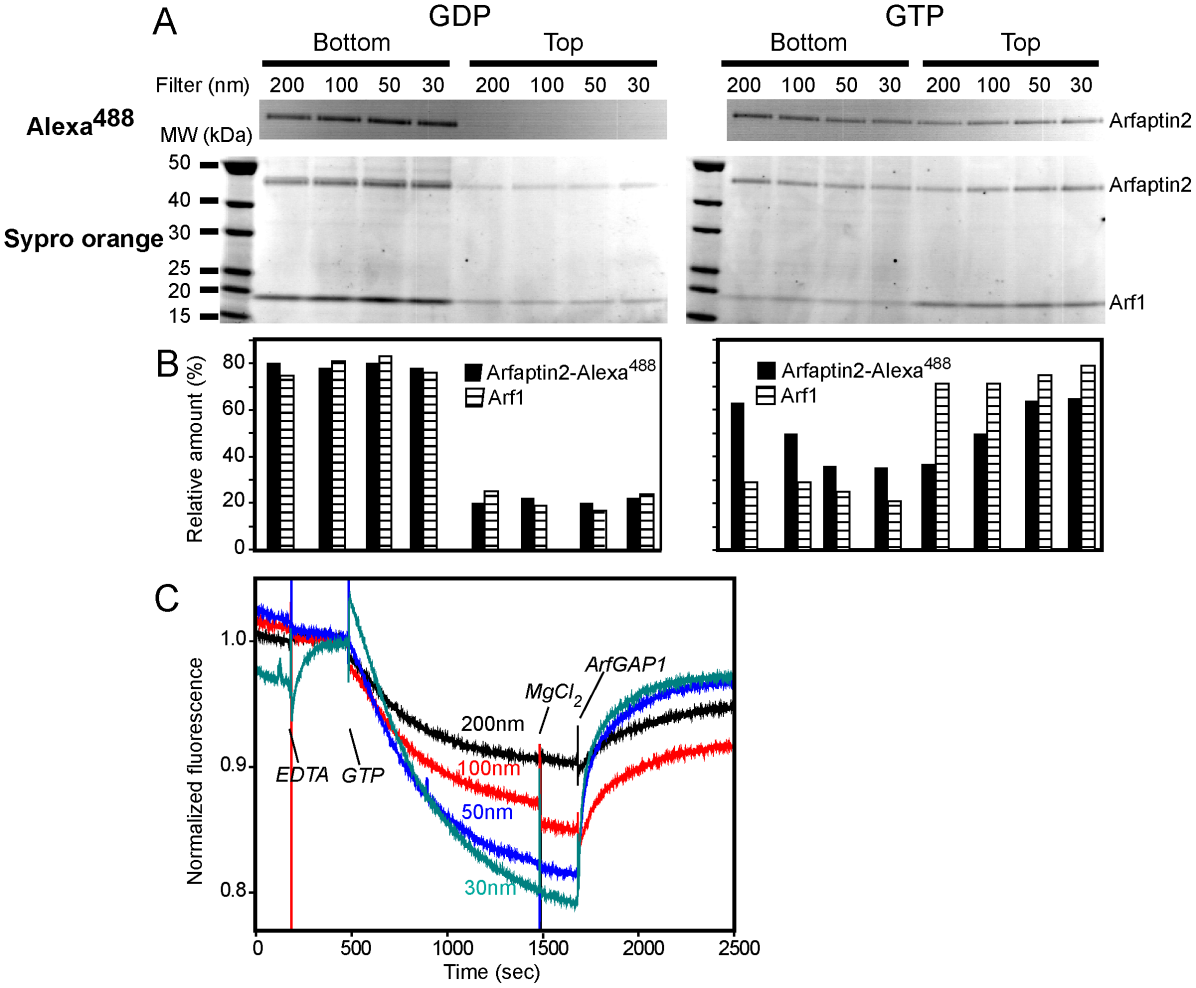
The binding of Arfaptin2 to liposomes requires Arf1 activation and increases with membrane curvature. A. Flotation assay. Arfaptin2 and Arf1:GDP (left) or Arf1:GTP (right) (0.75 µM) were co-incubated with Golgi-mix liposomes (0.75 mM lipids) extruded through filters with pores of decreasing sizes (200 nm, 100 nm, 50 nm and 30 nm). The suspension was adjusted to 30% w/v sucrose and overlaid with two cushions of decreasing sucrose density. After centrifugation, the top (liposomes) and bottom (unbound proteins) fractions were collected and analysed by SDS-PAGE. Direct Alexa^488^ fluorescence (top panels) was observed before Sypro-orange staining (bottom panels). B. Protein quantification from the Sypro-orange stained gels shown in A. The black bars correspond to Arfaptin2 and the white/black striped bars to Arf1. C. FRET assay. Arfaptin2-Alexa^488^ (0.5 µM) and myr-Arf1-H6 were mixed with Golgi-mix liposomes of various sizes containing 1 mol% TRITC-DHPE. At the indicated times, GTP (0.1 mM), EDTA (2 mM), MgCl2 (2 mM) and ArfGAP1 (10 nM) were added. Alexa^488^ fluorescence was followed in real time. Arfaptin2 was recruited to the membranes after Arf1 activation (GTP). Arfaptin2 recruitment increased as a function of membrane curvature.

To assess binding of Arfaptin2 to membranes of different curvatures and the role of Arf1 activation, we used two complementary assays: flotation on sucrose gradients and FRET. These experiments were performed on LUVs (Large Unilamellar Vesicles) of lipid composition mimicking that of the Golgi apparatus [24], [26]. For the flotation assay (Figures 1A and 1B), LUVs sequentially extruded through filters with pore sizes ranging from 200 nm to 30 nm were incubated with Arfaptin2 labelled with Alexa^488^ (Arfaptin2-Alexa^488^) in the presence of Arf1:GDP or Arf1:GTP. The suspension was then adjusted to a high sucrose concentration and overlaid with cushions of decreasing sucrose concentration. The bottom and top fractions were collected after ultracentrifugation. The LUVs are recovered in the top fraction [24], [26]. If Arfaptin2 binds to the LUVs, it will also be recovered in this fraction. When Arf1 was incubated with GDP, both Arf1 and Arfaptin2 did not bind to the vesicles and remained in the bottom fraction (Figures 1A and 1B). In contrast, when Arf1 was activated after incubation with GTP, Arf1 was detected in the top fraction in a similar amount regardless of the size of the vesicles. On the other hand, the amount of Arfaptin2 recovered in the top fraction increased with the curvature of the liposomes.

In the FRET assay, we monitored the binding of Arfaptin2-Alexa^488^ to LUVs of different sizes containing the fluorescent lipid TRITC-DHPE. If Arfaptin2 binds to the vesicles after Arf1 activation, a decrease in the fluorescent signal of Alexa^488^ should be observed because of FRET between the TRITC and Alexa^488^ fluorophores. Experiments shown in Figure 1C confirmed the observations from the flotation assay. After Arf1 activation, the FRET signal increased with LUV curvature. Interestingly, the binding of Arfaptin2-Alexa^488^ to TRITC-LUVs was reversible, as the addition of a catalytic amount of ArfGAP1 (10 nM), which induces the hydrolysis of GTP to GDP in Arf1, resulted in the recovery of Alexa^488^ fluorescence. As previously reported [24], the activity of ArfGAP1 was greater on high-curvature liposomes than low-curvature liposomes.

Of note, the effect of membrane curvature on Arf1-mediated Arfaptin2 binding was better detected in the FRET assay than in the flotation assay. This could be due to the following reasons. In the flotation assay, both bound and unbound proteins are detected whereas the FRET assay only monitors membrane-bound Arfaptin2. Protein precipitation may also occur during the flotation assay that could contaminate the bottom fraction. Finally, about 20% of Arfaptin2 is recovered in the top fraction even in the presence of Arf1:GDP.

All together, the above data indicate that Arfaptin2 is sensitive to membrane curvature when recruited by Arf1:GTP. The Arf1-Arfaptin2 interaction is GTP-dependent and can be reversed upon GTP hydrolysis.

### Arfaptin2 is Preferentially Recruited on Membrane Tubes after Interaction with Arf1:GTP

**Figure 2:**
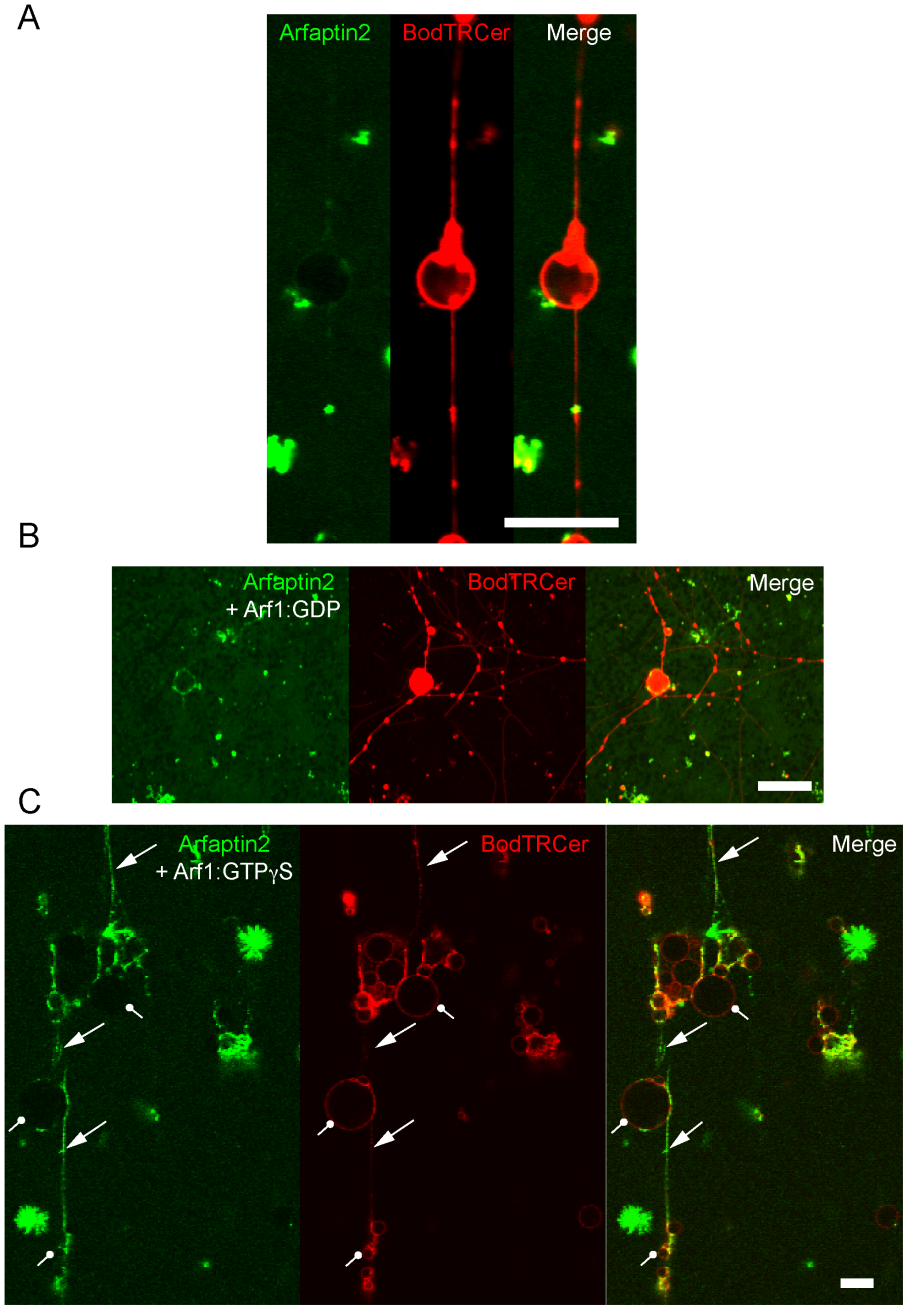
Arfaptin2 binds preferentially to membrane tubes connected to GUVs when Arf1 is in its active GTP-bound state. Membrane tube networks were pulled from Golgi-mix GUVs containing 1% red fluorescent lipid BodTRCer and 1% biotinylated lipid Biot-CAP-PE by a truncated biotinylated version of kinesin1. Tube networks (red panel) were incubated with (A) 1 µM Arfaptin2-Alexa^488^, (B) 1 µM Arfaptin2-Alexa^488^ and 1 µM Arf1:GDP, or (C) 1 µM Arfaptin2-Alexa^488^ and 1 µM Arf1:GTPγS. Arrows ending with a triangle point to highly curved membrane regions (tubes) where Arfaptin2 is bound. Arrows ending with a circle point to weakly curved membrane regions (vesicles) where Arfaptin2 binding is not detected. Scale bar: 10 µm.

We next monitored the binding of Arfaptin2 to membranes that displays flat and curved regions in continuity. This was achieved by pulling membrane tubes from giant unilamellar vesicles (GUVs) using molecular motors [19], [27]. We previously found that Arf1:GTP binding is only weakly sensitive to membrane curvature and binds to both GUV (flat membrane) and tubes (curved membrane) [19]. As shown in Figure 2 (A and B), Arfaptin2-Alexa^488^ alone or in the presence of Arf1:GDP did not bind to either GUV or tube membranes. In contrast, when Arf1 was loaded with the non-hydrolysable analogue of GTP, GTPγS, Arfaptin2-Alexa^488^ binding was observed in the tubular region, but not on the GUV membrane (Figure 2C). The calculation of the relative density of Arfaptin2 bound to GUV and tube membranes (distribution ratio given by (Arfaptin2-Alexa^488^/BodTRCer)_tube_/(Arfaptin2-Alexa^488^/BodTRCer)_GUV_; see [19]) gave a value close to 20, indicating that Arfaptin2 preferentially binds to tubular regions. These results confirm and extend those obtained with LUVs: Arf1:GTP preferentially recruits Arfaptin2 to highly curved membranes even under conditions where both proteins could interact on flat membranes.

### Arfaptin2 Induces Membrane Deformations When Recruited by Arf1:GTP at High Concentration

**Figure 3:**
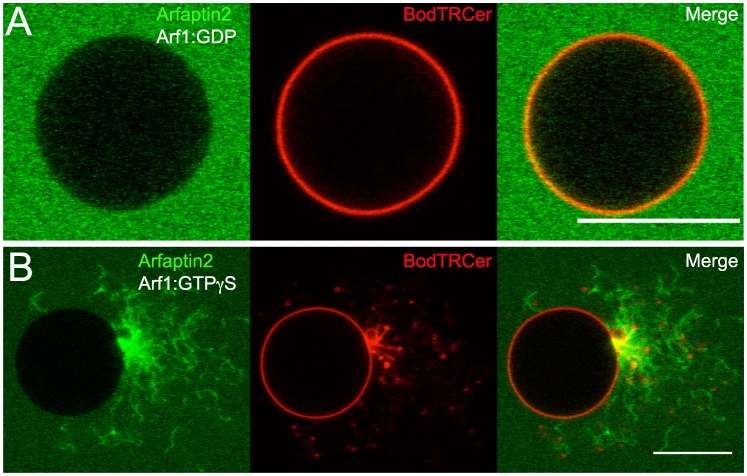
Arfaptin2 induces membrane tubulation at high concentration. Golgi-mix GUVs containing 1% of the red fluorescent lipid BodTRCer were incubated with (A) 3 µM Arfaptin2-Alexa^488^ and 3 µM Arf1:GDP or (B) 3 µM Arfaptin2-Alexa^488^ and 3 µM Arf1:GTPγS. Scale bar: 10 µm.

It has been shown that Arfaptin2 can tubulate liposomes *in vitro* [9] and induce the formation of membrane tubes when overexpressed in cells [13]. Under our experimental conditions, no membrane deformation was observed on GUVs incubated with Arf1:GTPγS and Arfaptin2-Alexa^488^ at concentrations below 1 µM (data not shown). However, tubular protrusions emerging from the vesicles could be seen at higher concentration (3 µM) in the presence of Arf1:GTP (Figure 3). As expected, no Arfaptin2 signal was detected on the GUV membrane, but only on tubular extensions.

In conclusion, our work illustrates a dual mechanism for recruiting Arfaptin-2 to liposomes: activation of Arf1 and membrane curvature. How could this work? A likely explanation is given by the recently solved crystal structure of the complex between Arl1 and the Arfaptin2 BAR domain [14]. In this study, Nakamura and coll. show that two Arl1 molecules bind to the edge of the Arfaptin2 BAR domain, leaving the concave part of this domain free to interact with a curved membrane. To our knowledge, no 3D structure of Arf1 in complex with Arfaptin2 is available. It has been proposed that Arf1 can compete for Rac1 binding to Arfaptin2 [4]. The Rac1-binding site is located in the central region of the BAR crescent shape [4], which then would weaken the above hypothesis. However, the results of Nakamura and coll. suggest that, although one molecule of Rac1 and two molecules of Arl1 cannot coexist on the Arfaptin2 BAR domain, it is likely that one molecule of Rac1 and one molecule of Arf1 can associate. It will then be important to investigate whether Arf1 binds to Arfaptin2 at the same sites as Arl1 and to perform competitive binding experiments on membranes.

The exact biological function of the Arf1-Arfaptin2 interaction is not known. Although Arf1 is present on Golgi membranes, Arfaptin2 appears to be recruited on these membranes by Arl1, but not by Arf1 [13]. In any case, our results point to a generic mechanism that could be used by both Arf1 and Arl1 in the formation of transport vesicles: Arfaptin2 is preferentially recruited to the nascent vesicle (curved membrane) where it interacts with other proteins involved in membrane deformation and/or fission. Hydrolysis of GTP-bound Arl1/Arf1 could then promote its dissociation.
